# In this issue: Volume 115, Issue 9, September 2024

**DOI:** 10.1111/cas.16328

**Published:** 2024-09-06

## FOXM1 and DEPDC1 feedback loop promotes hepatocarcinogenesis and represents promising targets for cancer therapy

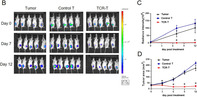

One of the most common characteristics of cancer cells is their ability to proliferate (multiply) in an uncontrolled manner. Liver cancer is one of the major causes of cancer‐related death worldwide. Understanding the processes which lead to abnormal cancer cell multiplication can help improve treatment approaches for liver cancer, and can even lead to the development of new anticancer drugs.

The *Forkhead Box M1* gene, or *FOXM1*, is known to be involved in cancer cell proliferation. However, the exact mechanism behind this is unclear. In this study, Wei et al. have shown that the interaction between *FOXM1* and the gene *DEPDC1* (*DEP domain containing 1*) may play a crucial role in the development of hepatocellular carcinoma (HCC), which is the most common type of liver cancer.

Their studies of genetically modified HCC cells showed that these genes interacted with each other in a ‘positive feedback’ loop, wherein *FOXM1* required the presence of *DEPDC1* to carry out its cellular activities, and vice versa. Most importantly, this interaction was shown to be vital for cancer cell growth – inactivating *DEPDC1* was found to reduce tumor growth in mice with liver cancer. Studies of human HCC samples confirmed these results, showing that the *FOXM1/DEPDC1* interaction was crucial in the development of liver cancer.

The next step was to study whether *FOXM1* or *DEPDC1* could be targeted to treat liver cancer using immunotherapy approaches. Immunotherapy is a kind of cancer treatment that helps a patient's own immune system act against tumors. In this case, the authors were successful in generating T‐cells (a type of immune cell) that could specifically target the FOXM1 or DEPDC1 proteins. These ‘engineered’ T‐cells showed tumor‐killing abilities in cell cultures as well as in mice.

Overall, these results suggest that the interaction between FOXM1 and DEPDC1 is very important for the proliferation of cancer cells in HCC. Targeting them using immunotherapy‐based approaches could be a potential future strategy for the treatment of liver cancer.

https://onlinelibrary.wiley.com/doi/10.1111/CAS.16273

## Tumor eradication by triplet therapy with BRAF inhibitor, TLR 7 agonist, and PD‐1 antibody for *BRAF*‐mutated melanoma

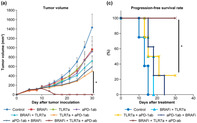

Melanoma is a notoriously difficult cancer to treat, with *BRAF*‐mutated melanomas representing one of its most challenging forms. Although BRAF and MEK inhibitors are effective for this form of melanoma, drug resistance occurs. Therefore, combinations of BRAF inhibitors and immune checkpoint inhibitors have been attempted, but they were not so effective, highlighting the urgent need for more innovative strategies. This resistance is partly driven by mechanisms such as the production of immunosuppressive molecules, the exhaustion of antitumor T cells and the insufficient innate immune responses. In response to this challenge, Nakamura et al. developed an innovative combination aimed at tackling the complex ways in which *BRAF*‐mutated melanoma evades the immune system. The team designed a three‐part treatment approach, testing it on mice implanted with human or murine melanoma cells carrying the *BRAF* mutation.

The first component of the therapy is vemurafenib, a drug that specifically targets and inhibits the *BRAF* gene's activity, which is crucial for the cancer cells' growth. By blocking this pathway, vemurafenib also reduces the production of molecules that the melanoma cells use to suppress the immune system.

The second component is an anti‐PD‐1 antibody. PD‐1 is a protein that helps cancer cells hide from the immune system. By blocking this protein, the anti‐PD‐1 antibody helps the body's immune cells recognize and fight the cancer more effectively.

The last component is a drug called DSR6434, a Toll‐Like Receptor 7 (TLR7) agonist. This drug boosts the body's innate immune system—its first line of defense—by activating specific immune cells like plasmacytoid dendritic cells (pDCs) and natural killer cells, subsequently intratumoral conventional dendritic cells (cDCs) and T cells. These cells play a crucial role in boosting a strong immune response against the cancer.

The results were compelling. The combination therapy outperformed single or double treatments by effectively fighting the tumor. It significantly boosted the activity of T‐cells, through inhibiting immunosuppression and stimulating innate immunity. In mouse models, this therapy led to notable tumor shrinkage, overcoming the cancer's ability to hide from the immune system. These findings suggest that the combined use of BRAF inhibitors, PD‐1 blockers, and TLR7 agonists offers a powerful new approach to treat *BRAF*‐mutated melanoma. This method targets both tumor growth and immune resistance, potentially offering a more lasting solution.

https://onlinelibrary.wiley.com/doi/10.1111/CAS.16251

## Prostate cancer cancer‐associated fibroblasts with stable markers post‐androgen deprivation therapy associated with tumor progression and castration resistant prostate cancer

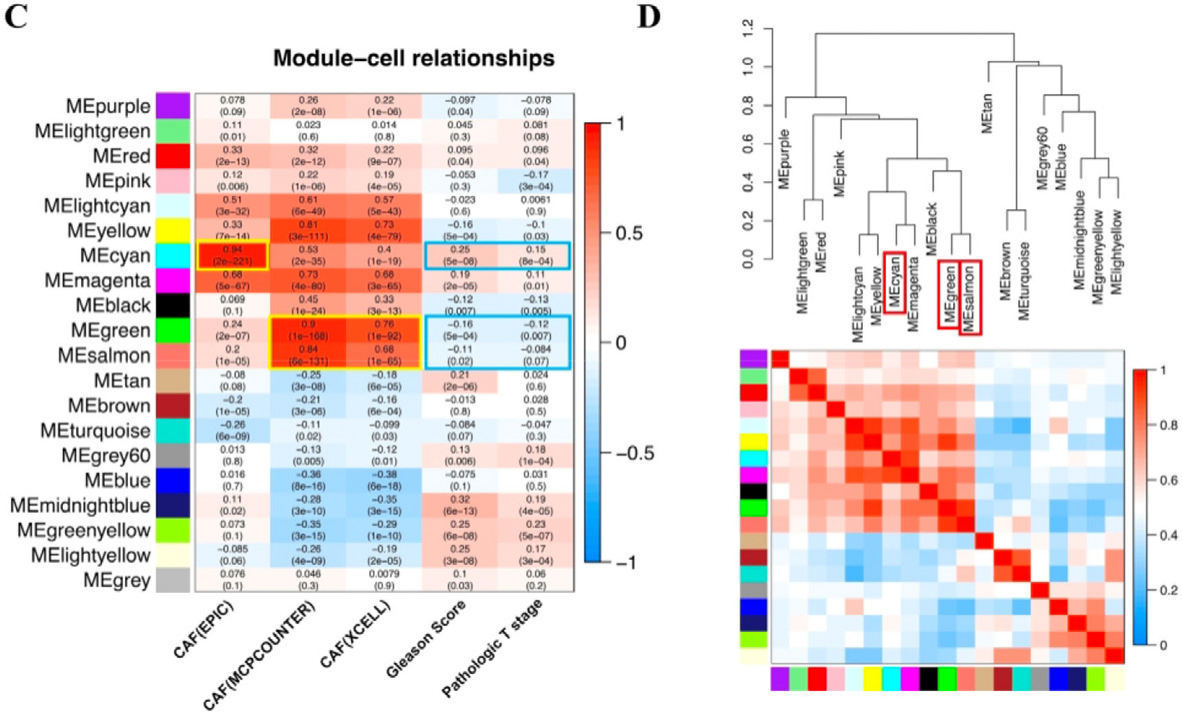

Prostate cancer (PCa) is a serious health concern among men due to its rising prevalence and high mortality rates. Testosterone plays a key role in PCa development, leading to treatments like androgen deprivation therapies (ADT) that aim to lower testosterone levels. While initially effective, these interventions often lead to the development of metastatic castration‐resistant PCa (mCRPC), an advanced form of PCa, where testosterone‐targeting therapies become ineffective. Previous research has suggested that cancer‐associated fibroblasts (CAFs), a cell type promoting tumorigenesis in the tumor microenvironment, may contribute to the progression of PCa and castration‐resistant prostate cancer (CRPC). However, studies investigating this link are limited.

To address this gap, Pan et al. conducted a study to establish the clinical significance of CAFs in PCa and identify PCa‐specific CAF gene signature, i.e., a group of genes involved in PCa progression. For this, they obtained PCa and CAF cell line data and used weighted gene co‐expression network analysis to determine a unique PCa‐specific gene signature in CAFs. The specificity of the signature was validated through techniques like single‐cell RNA sequencing, cell line RNA sequencing, and immunohistochemistry.

To investigate the effects and status of CAFs following ADT treatment, the researchers compared non‐ADT samples with those taken one‐month post‐ADT.

They observed that the specific signature and highest signal output state of CAFs remained unchanged, indicating that these cells play a crucial role in stabilizing the tumor microenvironment and are not influenced by ADT treatment.

In conclusion, the study effectively identified a distinct signature for CAFs in PCa. It showed that CAFs are linked to PCa progression, increased Gleason scores, and the development of CRPC. The importance of CAFs in PCa was underscored by their consistent high signal output after ADT and the stability of their gene signature. These findings lay the groundwork for further CAF research in PCa and guide future studies.

https://onlinelibrary.wiley.com/doi/10.1111/CAS.16267

